# Nutcracker Syndrome Revealed by Hematuria in a Young Woman: A Case Report and Literature Review

**DOI:** 10.7759/cureus.63405

**Published:** 2024-06-28

**Authors:** Lotfi Majdi, Aziz Miftah, Youssef Janati, Youness Ait Bella

**Affiliations:** 1 Radiology, Multidisciplinary Hospital, Khouribga, MAR; 2 Nephrology, Multidisciplinary Hospital, Khouribga, MAR; 3 Urology, Multidisciplinary Hospital, Khouribga, MAR; 4 Intensive Care Unit, Multidisciplinary Hospital, Khouribga, MAR

**Keywords:** literature review, diagnosis, abdominal pain, nutcracker syndrome, case report, hematuria

## Abstract

Nutcracker syndrome is a rare condition that arises from the left renal vein getting compressed between the aorta and the superior mesenteric artery. Despite its clinical significance, this syndrome is often challenging to diagnose and is frequently overlooked. Its key clinical presentations include hematuria and pelvic or back pain. This condition involves elevated pressure on the left renal vein, leading to various signs and symptoms, with hematuria being a common manifestation. Herein, we report a 28-year-old woman with no medical history who presented with hematuria for two months. Abdominal CT revealed compression of the left renal vein between the superior mesenteric artery and abdominal aorta, with dilated left ovarian vein and pelvic varices, consistent with nutcracker syndrome.

## Introduction

Nutcracker syndrome (NS) is an uncommon condition that results from the left renal vein being compressed between the aorta and the superior mesenteric artery, leading to reduced blood outflow [1,2]. The syndrome mainly presents with hematuria and pelvic or back pain, predominantly affecting females aged 20 to 40 years [3]. Other symptoms include gonadal vein syndrome, varicocele, and proteinuria, as well as nonspecific gastrointestinal symptoms like nausea and appetite loss [4]. Diagnosis is often challenging and under-recognized. The prevalence of NS is uncertain due to symptom variation and lack of standardized diagnostic criteria [5,6]. It's crucial to differentiate NS from the nutcracker phenomenon, which describes the anatomical or radiological finding of left renal vein compression without symptoms [7]. Treatment protocols are unclear. Advances in imaging have improved diagnosis, and endovascular surgery has become a less invasive treatment option compared to the past, when open surgical procedures were the norm [1]. In this report, we describe a 28-year-old woman with no prior medical issues who experienced hematuria for two months. A CT scan showed the left renal vein compressed between the superior mesenteric artery and abdominal aorta, along with a dilated left ovarian vein and pelvic varices, indicating nutcracker syndrome.

## Case presentation

**Figure 1:**
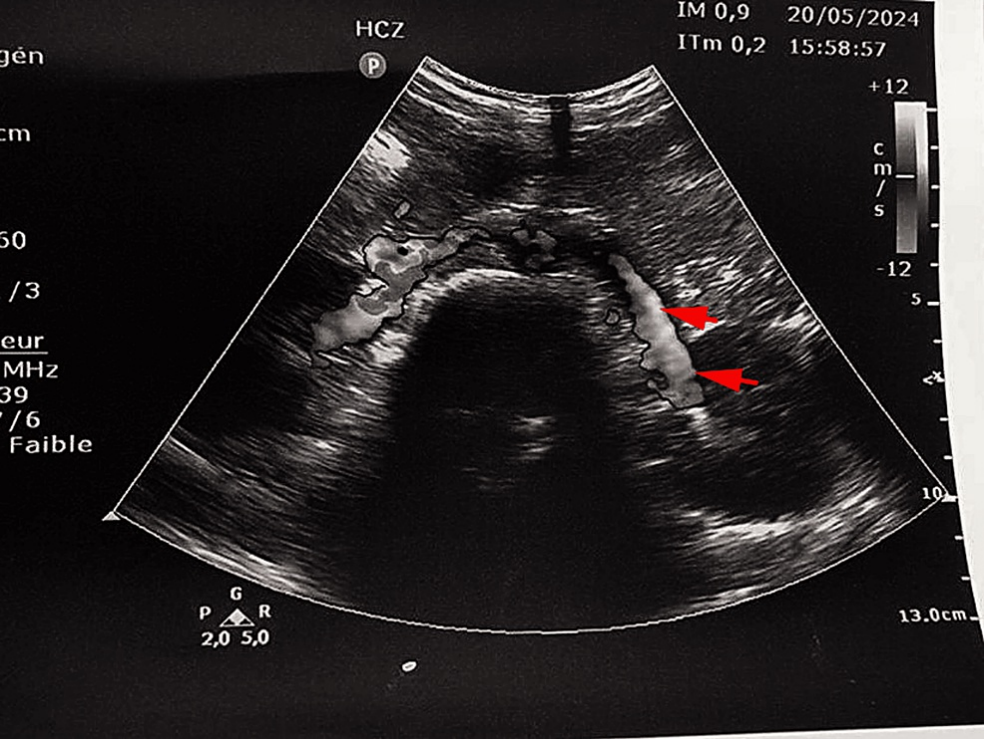
Ultrasound of the abdomen An abdominal ultrasound revealed ectasia of the left renal vein (red arrows).

We report the case of a 28-year-old female patient with no particular medical history. She was admitted for hematuria lasting two months, initially of low abundance then becoming more abundant. She also complained of mild abdominal pain in the lower abdomen. No digestive symptoms, such as nausea or vomiting have been reported by the patient. The hematuria was intermittent and, at times, macroscopic with clots. The patient does not smoke and received no medical treatment prior to her admission. The patient denied having previous hematuria episodes, gynecologic complaints, nephrology disease, diabetes, or a family history of hematuria. Physical examination revealed normal vital signs. Deep palpation pain was reported by the patient, without signs of peritoneal irritation. The urinalysis results were as follows: leukocytes at 2.104 leukocytes /ml, erythrocytes at 6.105 red blood cells/ml, and protein and nitrite were both negative. Other laboratory tests, including renal function and blood count showed no changes. A total abdominal ultrasound revealed ectasia of the uterine vessels (Figure 1).

**Figure 2:**
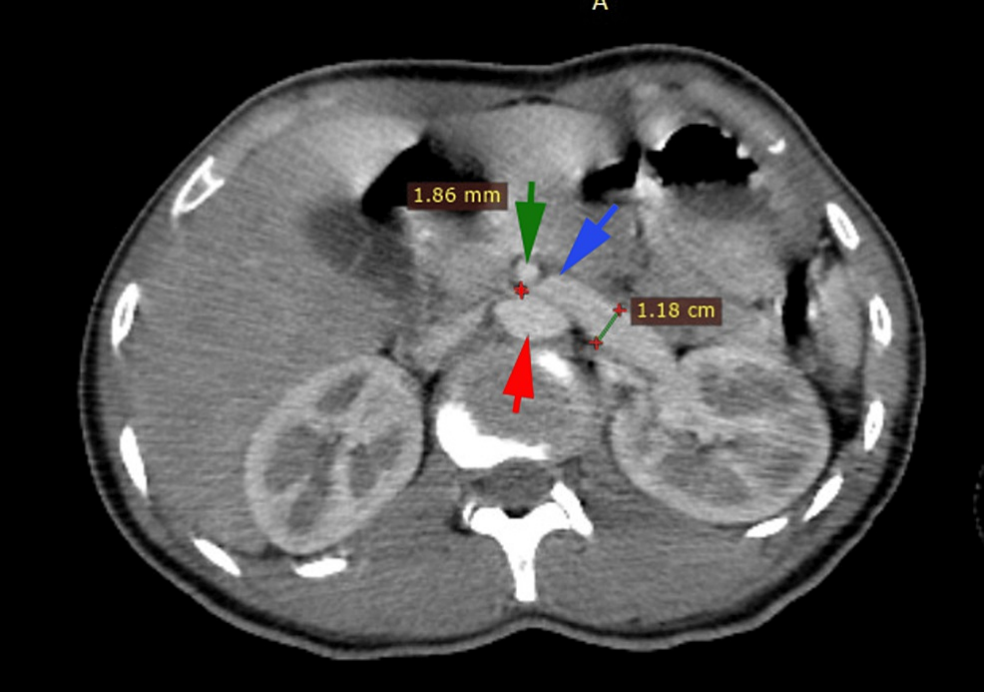
Abdominal CT scan image Tomography image demonstrating the compression of the left renal vein (blue arrow) by the aorta (red arrow) and the superior mesenteric artery (green arrow).

**Figure 3:**
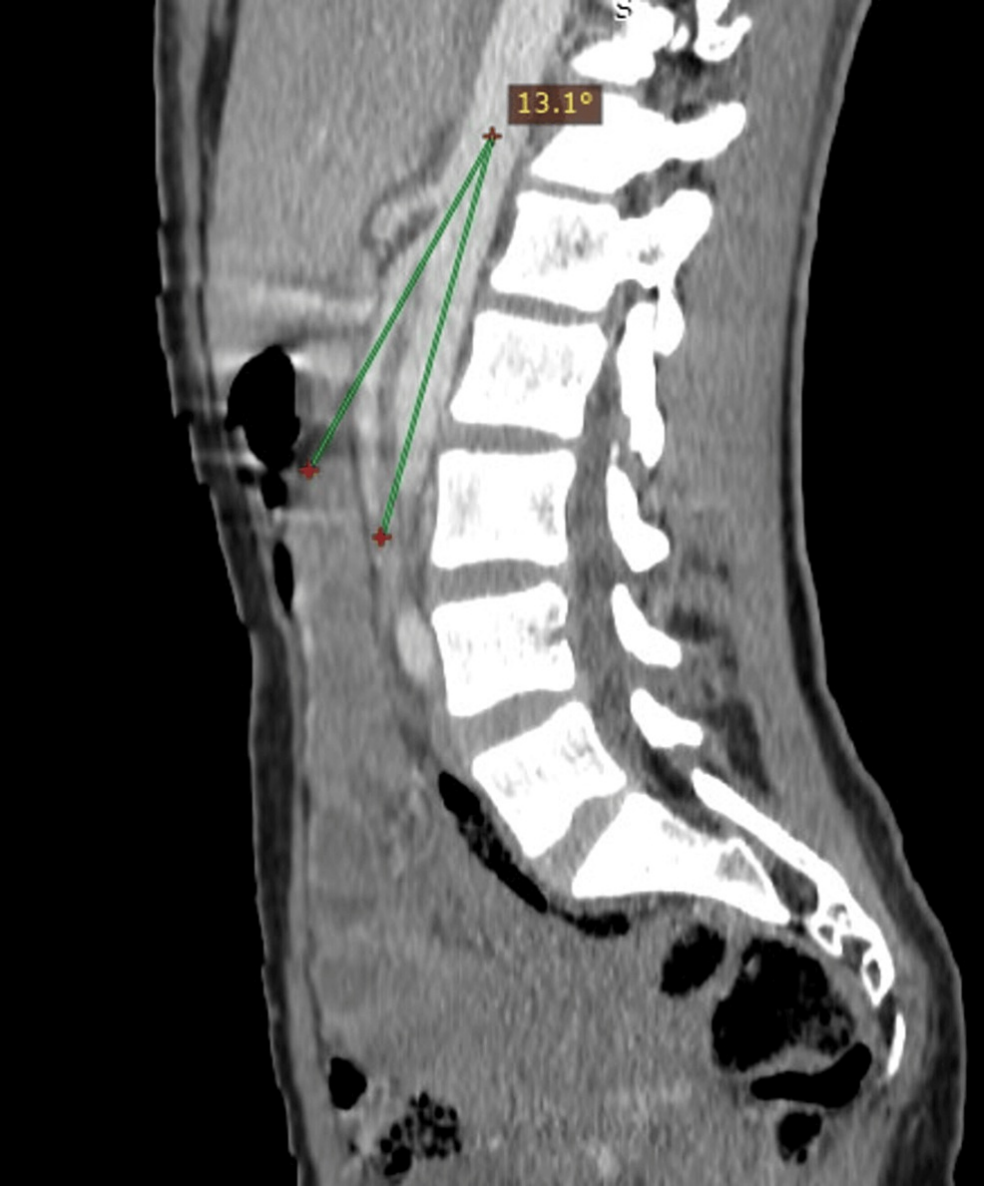
CT scan of the abdomen and pelvis (sagittal view) The image shows the angle between the abdominal aorta and the superior mesenteric artery which is measured at 13.1° (less than 41°), meeting the diagnostic criteria for Nutcracker Syndrome (NCS).

**Figure 4:**
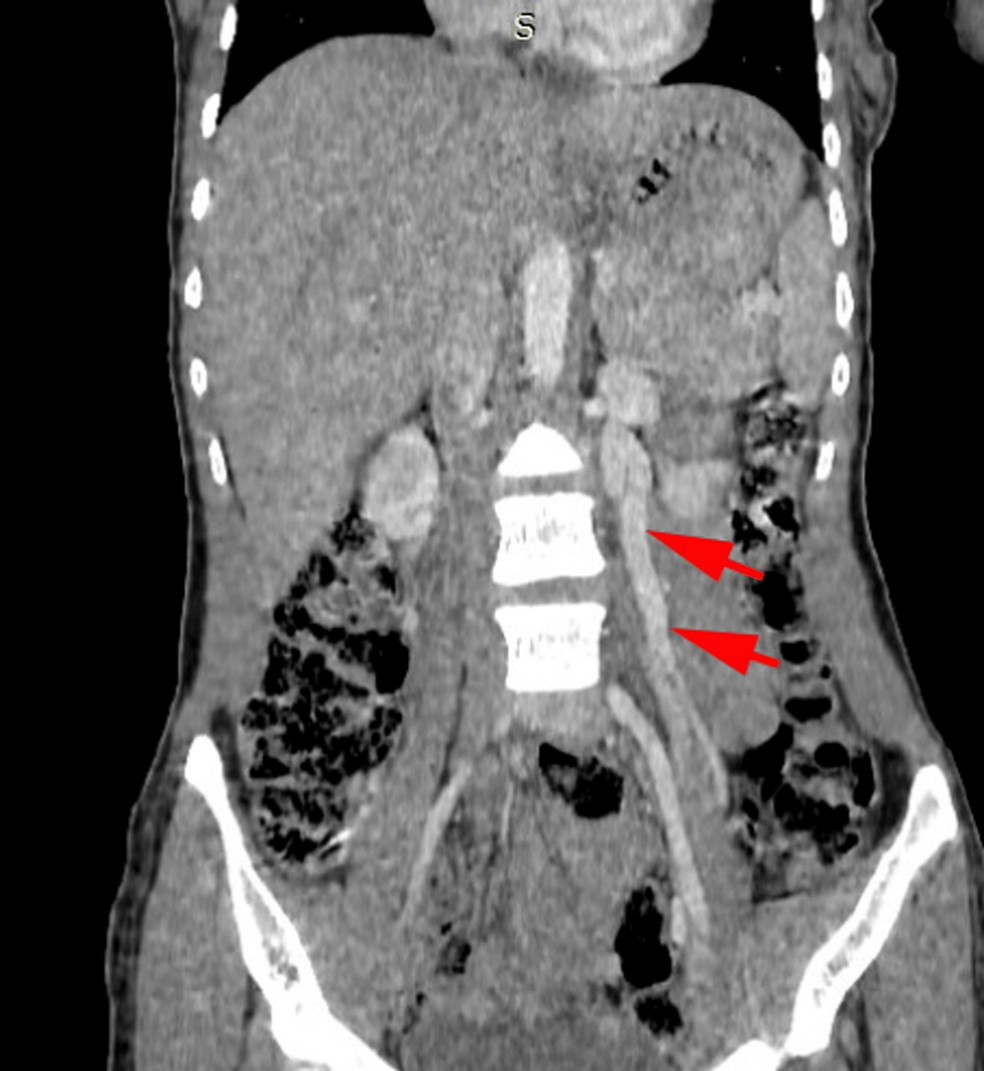
CT scan of the abdomen and pelvis (coronal view) The image is showing dilation of the left ovarian vein (red arrows).

**Figure 5:**
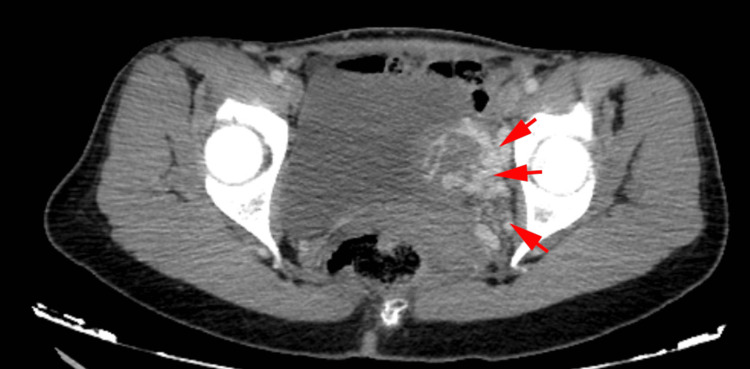
CT scan of the pelvis (axial view) The red arrows are showing pelvic varices.

Abdominal CT scan with intravenous contrast revealed extrinsic compression of the left renal vein between the superior mesenteric artery and the abdominal aorta posteriorly (Figures 2, 3), with tortuous dilatation of the left ovarian vein (Figure 4), associated with significant peri-uterine bilateral pelvic varices (Figure 5).

The patient was hospitalized for one week to manage pain and assess the need for vascular surgery. The patient continued to receive outpatient care and was scheduled for a surgical procedure.

## Discussion

Initially described by El-Sadr and later named by Schepper [8], Nutcracker syndrome (NS) involves compression of the left renal vein between the aorta and superior mesenteric artery, with a higher prevalence in women [1]. Nutcracker syndrome (NS) is often underdiagnosed, with anatomical changes producing no specific symptoms [8]. D’Archambeau et al. reported an 83% incidence of the syndrome in patients with pelvic congestion [9]. On the pathophysiological level, It is believed to be associated with nephroptosis or reduced retroperitoneal fat, leading to left renal vein (LRV) elongation and a reduced angle between the superior mesenteric artery (SMA) and aorta. Various symptoms have been reported in NS, such as flank pain, hematuria, left varicocele, fatigue, proteinuria, and pelvic congestion. Hematuria, as in our case, can result from the rupture of small septa separating veins in the urinary collecting system [10]. Pain in NS is multifactorial and may have a hormonal component, as it worsens during the premenstrual period when progesterone induces vasodilation and increases blood flow [11]. Due to its nonspecific symptoms, NS can be difficult to diagnose and is often mistaken for other conditions, particularly nephrolithiasis. Diagnosis typically occurs after excluding other diseases [12]. The diagnosis of NS is confirmed when the reduction in the diameter of the left renal vein is greater than 50% [13]. Vascular Doppler ultrasound can also aid in diagnosis, although it was not performed in this case. Treatment for NS remains controversial and should be based on individual patient characteristics and severity of symptoms. Options vary from conservative observation to endovascular stenting (EVS) or open surgery, depending on symptom severity and local expertise [1]. Conservative treatment may be attempted for mild symptoms, while surgical intervention is indicated for severe cases with anemia, significant pain unresponsive to analgesics, or renal impairment [14]. Estrogens and anti-inflammatory drugs have been used in clinical trials with limited success [15]. Surgical options include left renal vein transposition, renal autotransplantation, and ligation of the ovarian vein and pelvic varicose veins [9]. Endovenous intervention, offering a minimally invasive approach, is increasingly favored as the primary treatment for symptomatic cases. This shift is partly due to the successful treatment outcomes seen in iliac vein compression conditions, such as May-Thurner syndrome, which closely resembles NCS [16]. The six largest retrospective studies, covering a total of 192 endovascular stenting-treated patients [16], report symptom outcomes. Most patients in these studies experienced complete or partial symptomatic improvement following the procedure [16]. The most concerning endovascular stenting complication is stent migration. Wu et al. [17] reported stent migration in five out of 75 patients (6.7%) over an average 55-month follow-up period.

## Conclusions

The diagnosis of nutcracker syndrome often requires excluding other causes of chronic pelvic pain and hematuria including interstitial cystitis, pelvic inflammatory disease, endometriosis, pelvic tumors, or inflammatory bowel disease. NS requires a high level of suspicion for diagnosis. Endovascular treatment is currently one of the new treatment standards and has very few limitations.
